# Gut Dysbiosis: A Target for Protective Interventions against Parkinson’s Disease

**DOI:** 10.3390/microorganisms11040880

**Published:** 2023-03-29

**Authors:** Illyane S. Lima, Ana C. Pêgo, Ana C. Martins, Ana R. Prada, João Tomás Barros, Gracelino Martins, Raffaella Gozzelino

**Affiliations:** NOVA Medical School Research, NOVA University of Lisbon, 1150-082 Lisbon, Portugal

**Keywords:** gut microbiome, inflammation, neuroinflammation, iron, Parkinson’s disease

## Abstract

Sub-chronic inflammation, caused by age-related dysbiosis, primes the brain to neuroinflammation and neurodegenerative diseases. Evidence revealed that Parkinson’s disease (PD) might originate in the gut, demonstrating gastro-intestinal disturbances, as reported by PD patients long before developing motor symptoms. In this study, we conducted comparative analyses in relatively young and old mice maintained in conventional or gnotobiotic conditions. We aimed to confirm that the effects induced by age-related dysbiosis, rather than aging itself, sensitize to PD onset. This hypothesis was confirmed in germ-free (GF) mice, which proved resistant to the pharmacological induction of PD, regardless of their age. Contrary to conventional animals, old GF mice did not develop an inflammatory phenotype or an accumulation of iron in the brain, two catalysts sensitizing to disease onset. The resistance of GF mice to PD is reverted when colonized with stool collected from conventional old animals, but not if receiving bacterial content from young mice. Hence, changes in gut microbiota composition are a risk factor for PD development and can be targeted preventively by iron chelators, shown to protect the brain from pro-inflammatory intestinal priming that sensitizes to neuroinflammation and the development of severe PD.

## 1. Introduction

Since the 19th century, advances in disease prevention doubled life expectancy [1], which average, in 2017, was 78.3 years in European countries. Predictions are that, by 2050, the number of individuals reaching this age worldwide will be three times higher [2]. According to the World Population Prospects of the United Nations, 1 in 6 persons will be over 65 years, totaling 2.1 billion people [3]. Individuals reaching the age of 80 will account for 426 million, and the incidence of age-related disorders will increase exponentially [4]. Hence, identifying targets for the development of new therapeutic strategies is of utmost importance to ameliorate the impact of these diseases on the life quality of affected individuals. In addition, the need to reduce the economic burden that a prolonged life causes on the public health sector encouraged investigations to focus on disease prevention and etiology [3].

Aging is a natural and complex process. It is a genetically encoded physiological phenomenon often associated with compromised cellular and tissue functioning. Advancing age increases the risk of developing a variety of non-communicable diseases, which include neurodegenerative pathologies. Among these is Parkinson’s disease (PD) [5].

PD is the second most prevalent neurodegenerative disorder worldwide, after Alzheimer’s disease, with its prevalence doubling in the past two decades. PD is still a major health concern due to the difficulty in providing a diagnosis for early treatment. This is a long-term, progressive, and multifactorial pathology, genetically or sporadically induced, which affects 2–3% of the global population over 65 years of age. Estimates indicate that over 8.5 million individuals suffered from PD in 2019, equivalent to an increase of 81% since 2000 [6]. According to the WHO, 329,000 patients died from PD in 2019, representing an increase of 100% in the past decades. However, the possibility of developing this neurodegenerative disease doubles in elderly aged above 75 years [7]. Studies on gender prevalence demonstrate that men are more susceptible to developing PD [8]. Women usually report fewer symptoms, which are often less severe [9].

PD is characterized by a progressive loss of dopaminergic neurons (DNs) in the substantia nigra pars compacta (SNpc) of the brain, which is often accompanied by the formation of alpha-synuclein (α-syn) aggregates within intracellular compartments. Those are referred to as Lewy bodies [10]. In addition to motor and non-motor symptoms, gastrointestinal (GI) manifestations, such as dysphagia, impaired gastric emptying, gastroparesis, and constipation, were shown to affect more than 80% of PD patients [11]. Studies revealed that GI dysfunction could be considered an early symptom, usually developed up to two decades before motor dysfunction, and, as such, significantly impact the quality of life of affected individuals [12]. The prevalence of GI complaints by PD patients highlighted the involvement of the autonomic nervous system and its enteric division. The importance of this bidirectional communication, referred to as the gut–brain axis, was revealed by showing that the production of α-syn aggregates in the gut might propagate to the brain in a prion-like manner through the vagus nerve [13]. In animal models, the pathophysiology of α-syn was found to strictly depend on gut microbiota, as intestinal pathogens contribute to clear this protein [14].

In recent years, emphasis has been given to investigations pointing out that disturbances in gut microbiota might precede GI manifestations [15,16,17,18], seeking to develop new targets for an etiological treatment against PD that might diminish PD incidence and provide easy access to the brain [19,20,21].

Estimates indicate that a vast number of microorganisms, between 10^13^ and 10^14^, reside in the intestine [22]. Their composition varies with aging, which reduces microbial diversity [23] and promotes changes in gut integrity. Microbial extravasation to systemic circulation might underly the sub-chronic low-grade inflammation characterizing aging and increase the risk of developing age-related disorders, as shown in mouse models of PD [24,25,26,27]. The impact of these discoveries on PD was revealed by showing that patients undergoing truncal vagotomy are significantly protected against the risk of developing this pathology [28]. Comparative analyses in PD patients confirmed the role of specific pathogens in promoting disease transmission [29,30,31,32,33,34] and accelerating the progression of motor deficits. Age-related dysbiosis is associated with systemic inflammation and disruption of iron (Fe) homeostasis [35,36], which are known to contribute to tissue damage and malfunction [37]. The involvement of Fe in PD is well established [38,39,40], and Fe chelators were used as a therapeutical strategy to prevent neuronal death and disease progression [41,42]. However, deferiprone treatment was shown to worsen disease symptoms in PD patients that did not receive dopaminergic therapy [43,44].

In this study, we conducted comparative analyses to address the impact of gut microbiota on priming the brain to neuroinflammation and PD development. The colonization of germ-free (GF) mice with stool samples collected from age-matched animals and maintained in a conventional specific pathogen-free (SPF) environment revealed that changes in microbial composition are a risk factor for PD onset. We demonstrate that while fecal transplant to GF mice drives inflammation and disruption of Fe homeostasis, the translocation of bacterial components from aged mice treated with Fe chelators reduces the risk of developing a severe PD. Our research confirms the need to develop therapeutic strategies that act peripherally and in a preventive manner aimed at reducing the susceptibility to develop PD rather than treating the symptoms induced by this pathology.

## 2. Materials and Methods

### 2.1. Mice

C57BL/6J mice were purchased from the Champalimaud Foundation and Instituto Gulbenkian de Ciência, Portugal, where animals were bred and maintained under SPF conditions. Experimental protocols were approved by the Ethics Committee of the Institutions above and by the Portuguese National Entity (Direcção Geral de Alimentação e Veterinária; 0421/000/000/2018), according to the Portuguese (Decreto-Lei 113/2013) and European (Directive 2010/63/EU) legislations. Experiments with GF mice were conducted at the Instituto Gulbenkian de Ciência.

All mice were maintained in cages (3–5 animals per cage), undisturbed, in an environmentally controlled room, in terms of temperature and humidity conditions, with a 12 h light/12 h dark cycle, and fed with standard diet and water at libitum. Animal care was taken to ensure that any mice exhibiting signs of suffering or distress were euthanized with CO_2_. This also included animals showing normal conditions of disruption and general ill-health signs, causing difficulty eating or drinking or moderate discomfort, pain, or distress. Severe forelimb, hindlimb incoordination, and/or locomotor disabilities constituted end-point criteria to sacrifice the animals used in our experiments. All symptoms were noted, registered, and discussed with the veterinarian of the animal facility.

The hypothermia caused by MPTP administration was prevented by using heating pad, to which mice were allocated before the procedure. MPTP-induced mice were then transferred into pre-heated cages, 2 h after the last injection. Animal cages were changed every 96 h after the procedure, and the mice’s well-being was monitored until experiment day 30.

Experiments with germ-free mice were conducted in a fully sterile environment, i.e., the Axenic/Gnoto Facility of the Gulbenkian Institute of Science, where animals are kept in rigid isolators since birth, equipped with an individual HEPA filter for air in flow. Mice handling was performed by transferring chambers and containers, equipped with the Double Door Rapid Transfer Port (DPTE^®^). A connecting system was used to introduce and remove equipment and other materials to and from the sterile isolators. The Airtight Sealed Positive Pressure Individually Ventilated Cages technology allows to safely carry out experimental procedures.

Fecal contents were collected at the same hours and from aged-matched donor mice fed with the same diet to restrict potential parameters influencing biological variability. The samples were homogenized in sterile phosphate-buffered saline (PBS 1x) and filtered twice. Fecal transplantation was performed by oral gavage, with the fecal content suspended in 200 μL of PBS 1x and administered once a day for 5 consecutive days.

### 2.2. PD Induction and Evaluation of Locomotor Dysfunction

PD was pharmacologically induced, according to the protocol described in [45]. Mice were injected with 1-methyl-4-phenyl-1,2,3,6-tetrapyridine (MPTP; Sigma-Aldrich Ref. No. M0896, Darmstadt, Germany) at a dose of 20 mg/kg (4 injections, i.p., 2 h apart). Mice were monitored daily for 30 days.

Locomotor dysfunction was assessed by placing mice upside down on a vertical pole and measuring how many seconds they required to descend [46]. A default time of performance of 120 s was set in case the severity of induced PD prevented mice from descending the pole.

### 2.3. Deferiprone Treatment in Mice

Mice were injected with deferiprone (DFP, Sigma Aldrich, Ref. No. 379409; Darmstadt, Germany) at a dose of 10 mg/kg (1 injection per day, i.p.). Mice were injected for 15 days before further experiments were conducted the day after.

### 2.4. Blood–Brain Barrier and Gut Permeability

Mice were injected intravenously (i.v.) with 0.1 mL of 2% Evans Blue (Sigma-Aldrich, Ref. No 2129; Darmstadt, Germany), dissolved in saline solution, and killed 1 h later. Brain samples were harvested, weighed, placed in 2 mL of formamide, and left for 48 h at 37 °C, to extract Evans blue dye as described in [47]. Absorbance was measured at λ = 620 nm (Bio Rad SmartSpec 3000). A standard curve with fixed concentrations of Evans blue was used to calculate dye extravasation into the brain. Data are expressed as mg of Evans blue per g of brain tissue, as means ± standard deviation.

### 2.5. Iron Measurement

Brain samples were dried (24 h, 99 °C), dissolved (3M hydrochloric acid, HCl, 10% trichloroacetic acid; TCA; overnight at 65 °C), and diluted (10 µL) in water (590 µL). β-mercaptoethanol (10 µL), sodium acetate (pH 4.5; 500 µL) and bathophenanthroline-disulfonic acid (80 µL) were added to each sample (37 °C, 1 h). Absorbance was measured using a microplate reader (Bio-Rad 3550-UV) (λ = 53 m). Note that this assay measures primarily labile iron, although we cannot exclude residual heme detection.

### 2.6. Isolation of Brain-Infiltrated Immune Cells

Mice were sacrificed, perfused in toto (20 mL PBS), and brains were collected in HBSS, finely minced, and digested with collagenase VIII (0.2 mg/mL, Sigma-Aldrich, Ref. No C2139; Darmstadt, Germany) for 30 min at 37 °C. Brains were homogenized by passing samples through a 100 µM strainer and collected in 20 mL HBSS. Next, samples were centrifuged for 10 min at 1500 rpm at 4 °C. Supernatants were discarded, and brains were washed in 20 mL HBSS and centrifuged again for 10 min at 1500 rpm at 4 °C. To separate the infiltrated leukocyte fraction, samples were re-suspended in 10 mL of 30% Percoll gradient (GE Healthcare, Ref. No 17-0891-01; Darmstadt, Germany) and centrifuged for 20 min at 2500 rpm at room temperature, without break and acceleration. Myelin forming the upper layer was carefully removed, and brain-infiltrated leukocytes, precipitated at the bottom of the tube, were resuspended in 200 µL of PBS, centrifuged, and lysed for 5 min in 100 µL of red blood cells lysis buffer. Cells were then washed 3 times with 200 µL of PBS supplemented with 2% heat-inactivated FCS (Invitrogen) before being stained.

### 2.7. Flow Cytometry

The total number of infiltrated immune cells was measured by flow cytometry using a known concentration of reference 10 μm latex beads suspension (Polysciences Europe GmbH, Ref. No CC10N-10; Hirschberg an der Bergstrasse, Germany), co-acquired with a pre-established volume of cellular suspension. Dead cells were excluded by Propidium Iodide. Singlets were gated among live cells based on size and granularity. Cells were stained with Fc block (anti-CD16/CD32; BD Pharmingen™- Ref. No 553141; Madrid, Spain) to prevent non-specific binding for 20 min at room temperature. Cells were then washed in PBS supplemented with 2% heat-inactivated FCS and stained as follows. The presence of resident microglia (CD11b+CD45int) and CNS infiltrates, such as monocytes/macrophages (CD11b+CD45high), inflammatory monocytes/macrophages (CD11b+CD45highLy6Chigh), and helper and cytotoxic T cells (TCRβ+CD4+ and TCRβ+CD8+), was determined. Their activation was assessed by the expression of surface markers, such as CCR2 and MHC II, for microglia and monocytes/macrophages or CD44 and CD62L for T cells. Different cell populations, infiltrating the brain, were determined by using antibodies directly conjugated to PE, PE/Cy7, PercepCy5.5, APC, FITC, BV510, and/or APC/Cy7 (purchased by BD Biosciences, Franklin Lakes, NJ or eBioscience). Cells were analyzed on a FACSCanto II (BD Biosciences) cytometer. FACS data were analyzed with FlowJo V10.

Antibodies were purchased by BioLegend (CD11b anti-mouse, Ref. No. 101216; APC/Cy7 anti-mouse CD45, Ref. No. 103115; PE anti-mouse TCR β chain, Ref No. 109208; APC/Cy7 anti-mouse CD8, Ref. No. 100714; Pacific Blue anti-mouse MHC II, Ref. No. 107620; APC anti-mouse CD44, Ref. No. 103012; PE/Cy7 anti-mouse CD62L, Ref. No. 10441; Amsterdam, The Netherlands), BD Biosciences (FITC anti-mouse Ly-6C, Ref. No. 553104; Pacific Blue anti-mouse CD4, Ref No. 558107; Madrid, Spain), and R&D SYSTEMS (PE anti-mouse CCR2, Ref. No. FAB5538P; Abingdon, UK).

### 2.8. qRT-PCR

Total RNA was isolated from mouse brains, using TRIzol (GRISP, Ref. No GB23.0100; Porto, Portugal) and the RNeasy Mini Kit (Machery-Nagel; Dueren, Germany) from blood using the NucleoSpin^®^ RNA Blood (Machery-Nagel; Dueren, Germany) and from cell suspension using the NucleoSpin^®^ RNA XS (Machery-Nagel, Ref. No 12733391; Dueren, Germany). Total RNA was retrotranscribed to cDNA (Transcriptor First Strand cDNA Synthesis Kit, ThermoFisher LTI, Ref. No 18080-051; Bleiswijk, The Netherlands) for PCR with Power SYBR Green PCR master mix (BioRad, Ref. No 1725124; Madrid, Spain). Transcript number was calculated from the threshold cycle (Ct) of each gene with a 2−ΔΔCT method (relative number), normalized to ArbP0 or GADPH, and expressed as fold induction of animals used as controls. The primers used in this study are listed below in Table 1.

### 2.9. Calcein Measurement

Isolated cells were stained with Calcein AM Permeant Dye (ThermoFisher, R. No C1430; Bleiswijk, The Netherlands), which was added to the antibody mix and analyzed on a FACSCanto II (BD Biosciences) cytometer.

### 2.10. ELISA

Calprotectin was measured by ELISA according to manufacturer instructions (R&D Systems; Abingdon, UK).

### 2.11. Statistical Analysis

Locomotor dysfunction or flow cytometry experiments were performed between 2 to 3 times, using between 2 and 4 mice per time. The results were pooled and expressed as mean ± standard deviation to assess statistical differences. The total number of mice per condition varied and is indicated in figure legends. In the graphs, each dot represents a mouse. ELISA and qRT-PCR experiments were conducted twice when the results reproduced the same trend. Representative graphs are shown and expressed as mean ± standard deviation.

Statistically significant differences between two groups were assessed using a two-tailed unpaired Mann–Whitney test or t-test according to data distribution. Normal distributions were confirmed using the Kolmogorov–Smirnov test. Statistical differences between groups following a non-normal distribution were assessed by applying the Mann–Whitney. Comparisons between more than two groups were carried out by one-way ANOVA. Survival curves are represented by Kaplan–Meier plots, and the survival difference between the groups was compared using the log-rank test. No statistical method was used to predetermine the sample size. All statistical analyses were performed using GraphPad Prism 9 software. Differences were considered statistically significant at a *p* value < 0.05. NS: Not significant.

## 3. Results

### 3.1. Age-Related Dysbiosis Primes to the Development of PD

**Figure 1:**
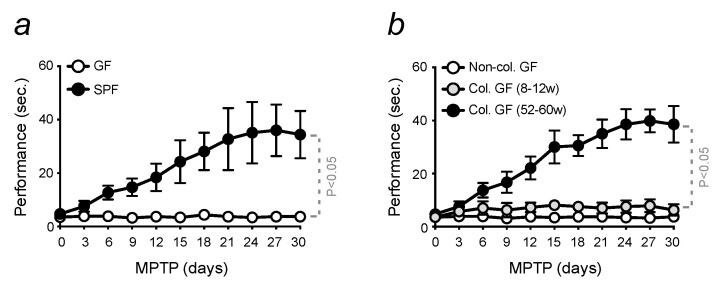
Age-related dysbiosis primes the development of PD. All mice are in C57BL/6 background (**a**) conventional (SPF) 8–12 weeks old (young) mice, and aged-matched GF animals were intoxicated with MPTP (20 mg/kg, i.p., 4 injections 2 h apart). Motor dysfunction was evaluated by assessing the time of performance on a pole test. Mice were monitored for 30 days after MPTP administration. (**b**) Motor performance of GF mice colonized or not (Ctrl) with stool samples collected from conventional young or 52–60 weeks old (old) mice, treated as in (**a**). The results are expressed as mean ± SD (*n* = 7–10 mice per group). Statistical analysis was performed by applying the one-way ANOVA test.

Pharmacological PD was induced by the intraperitoneal administration of MPTP, a neurotoxin capable of crossing the blood–brain barrier (BBB) and entering the brain, where converted into the active metabolite, MPP+. The selectivity of this compound for DNs was used to mimic the last phases of PD, i.e., neuronal loss. When C57BL/6 mice, aged between 8–12 weeks and referred to as “young”, were exposed to MPTP, they developed a severe locomotor dysfunction. Motor performance was evaluated using an established assessment of motor coordination, known as the pole test [46]. Mice were monitored for 30 days, starting upon MPTP administration. The sensitivity of animals maintained in a conventional SPF environment was reverted when the experiment was conducted with mice in gnotobiotic conditions. GF aged-matched C57BL/6 mice showed no motor symptoms in response to PD induction, as resistant to MPTP administration (Figure 1a). The lack of sensibility of GF mice to PD development was accompanied by the absence of a neuroinflammatory phenotype, which characterizes the disease in conventional mice. Infiltrating inflammatory cells, among which leukocytes, were found in the brain of PD-induced mice, and their activation was quantified by flow cytometry. An increased expression of the surface inflammatory marker, C-C chemokine receptor type 2 (CCR2), was detected in infiltrated leukocytes (Figure S1). CCR2 is known to act as a chemoattractant for the recruitment of additional monocytes/macrophages to the site of inflammation. In addition, an increased number of microglia was found in mice developing PD. Their activation, quantified by flow cytometry with the expression of the major histocompatibility complex II (MHCII) and CCR2, was shown to contribute to the neuroinflammatory phenotype caused by MPTP administration (Figure S2). Conversely, GF mice were resistant to neuroinflammation, as their profile resembled those of healthy mice. No difference in terms of the number of activated cells was observed in the brain of GF animals upon PD induction (Figures S1 and S2).

The resistance showed by GF mice induced with PD was reverted when colonized with stool collected from conventional 52–60 weeks old animals, referred to as “old”. As conventional mice, these mice started to develop locomotor dysfunction, which was assessed as a time of performance in descending the pole. When bacterial content is transferred into GF mice from stool collected from young animals, this effect was not observed. In this case, their motor function was not affected, indicating that the impact of age-related dysbiosis sensitized mice to PD and the development of severe symptoms (Figure 1b). Non-colonized mice, aged 8–12 weeks, were used as controls.

Overall, these data demonstrate that changes in gut microbiota, induced by aging, represent a risk factor for the development of PD, as priming the brain to disease onset.

### 3.2. Age-Related Dysbiosis Compromises Gut Integrity

**Figure 2:**
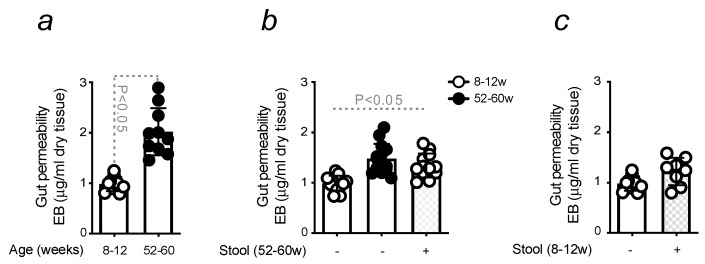
Age-related dysbiosis compromises gut integrity. (**a**) Gut permeability was measured by Evans Blue (EB) extravasation in conventional young or old mice. The effect of fecal microbiota transplantation is shown (**b**) in young GF mice colonized with conventional old stool or (**c**) with young samples. The results are expressed as mean ± SD (*n* = 7–10 mice per group). Statistical analysis was performed by applying the student’s t-test or one-way ANOVA.

The effects of age-related dysbiosis were investigated with the aim of better understanding their impact on the resistant phenotype showed by GF mice to PD induction. Hence, the role of gut microbiota in maintaining gut integrity [48] was confirmed by quantifying the extravasation of Evans Blue into the gut of conventional mice once injecting the dye into circulation. A significant increase in gut permeability was observed in old animals when compared to young mice (Figure 2a), which is consistent with previous studies published in the literature [35,49]. Disruption of gut integrity was evaluated in GF mice once colonized with stool collected from old conventional mice. Increased gut permeability was observed in these animals, and loss of gut integrity was comparable to conventional donors (Figure 2b). No significant changes in gut permeability were observed when stool from young animals was transferred into GF mice (Figure 2c).

Overall, these data demonstrate that age-related dysbiosis compromises gut integrity, increasing the permeability of the intestine.

### 3.3. Increased Gut Inflammation and Iron Content in Aged Mice

**Figure 3:**
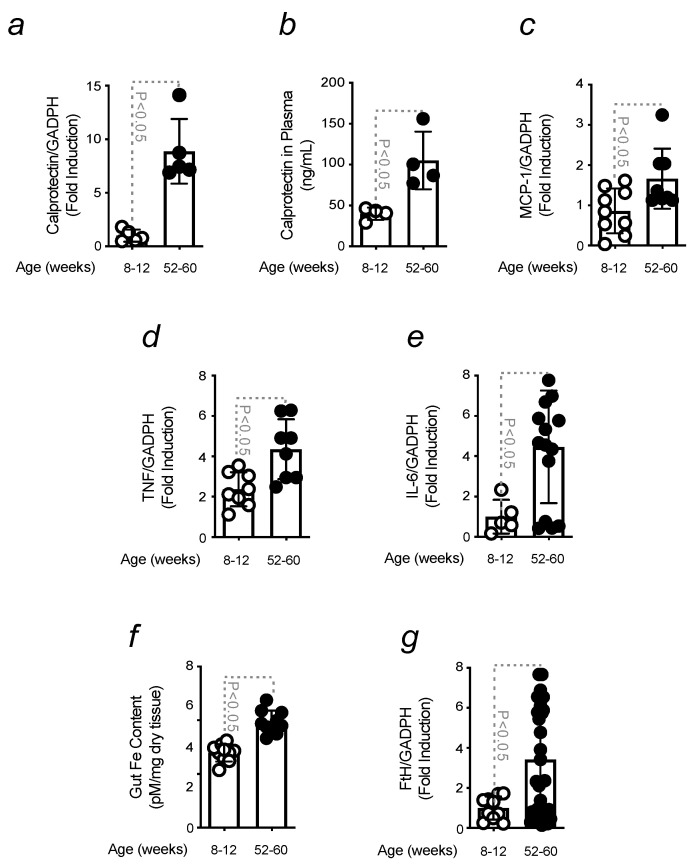
Increased inflammation and iron content in aged mice. (**a**) Calprotectin mRNA expression, quantified by qRT-PCR, in the gut of young vs. old mice, and (**b**) the results were confirmed by ELISA. mRNA expression of (**c**) MCP-1, (**d**) TNF, and (**e**) IL-6 as in (**a**). (**f**) Intracellular Fe content measured in the gut of mice as in (**a**). (**g**) mRNA expression of FtH as in (**a**). The results were normalized to Arbp0, used as housekeeping gene, and expressed as mean ± SD (*n* = 10–15 mice per group). The parametric Student’s *t*-test was applied to define statistical differences.

The inflammatory response caused by aging was assessed in the gut of old mice by qRT-PCR. Changes in the mRNA expression of inflammatory genes were quantified, and the results were compared to young mice. An increase in calprotectin was observed, during aging, in the gut of aged animals when compared to young (Figure 3a). Calprotectin is a calcium-binding protein released by activated inflammatory cells, which is used as a biomarker of intestinal inflammation [50]. The results were further confirmed by ELISA (Figure 3b). The expression of pro-inflammatory mediators, such as MCP-1, TNF, and IL-6, was also measured and found to increase in the gut of old mice in relation to young animals (Figure 3c–e). The data obtained are consistent with the recruitment of immune cells to the site of inflammation [51,52]. Promoting immune cell proliferation, Fe is essential for the establishment of a proper immune response [53]. Since aging is associated with an increased Fe level in different organs, we assessed whether that was also the case for the gut, given its role in dietary Fe absorption. A higher Fe content was observed in the gut of old mice when compared to young (Figure 3f). This result was further confirmed by the increased mRNA expression of the intracellular Fe-storing protein ferritin heavy chain (FtH) in the gut of old mice in relation to young animals (Figure 3g). Similar data were also obtained for divalent metal transporter 1 (DMT-1) and transferrin receptor 1 (TFR-1) expression (Figure S3a,b), these being genes involved in iron intake [54].

These results indicate that changes in gut permeability, occurring during aging, are associated with an inflammatory response, which contributes to the increased Fe content in the gut.

### 3.4. Increased Level of Intracellular Iron in Peripheral Blood Leukocytes during Aging

**Figure 4:**
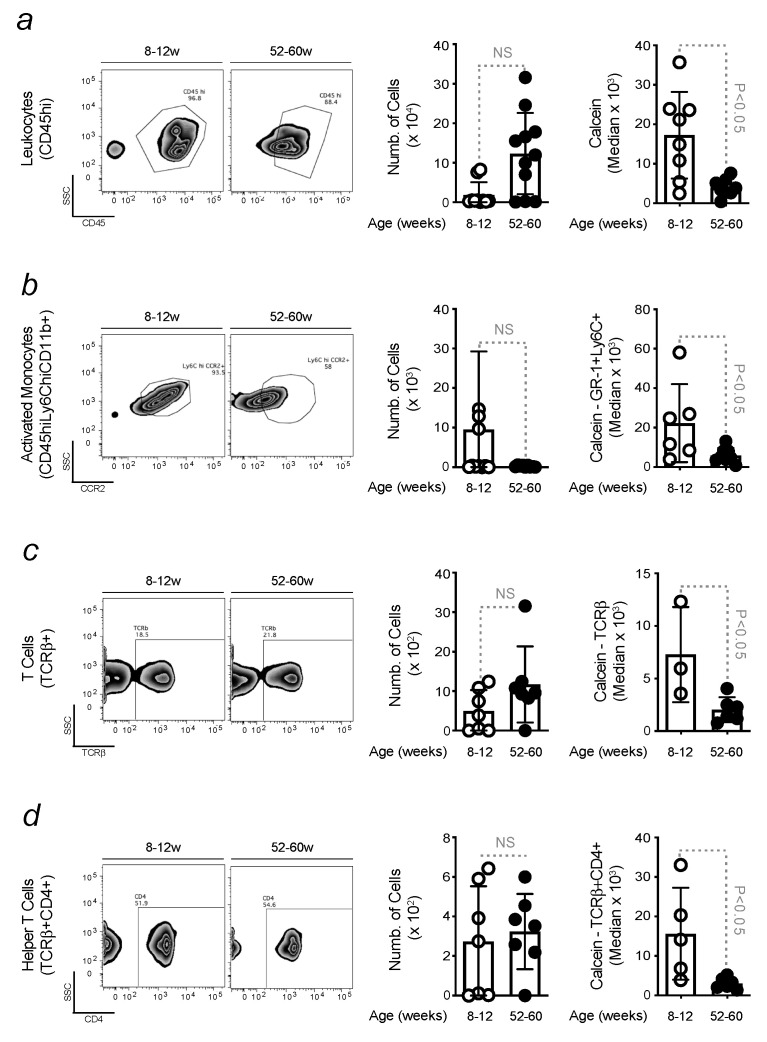
Increased level of intracellular Fe in peripheral blood leukocytes during aging. Gating strategy of (**a**) freshly isolated peripheral blood leukocytes (CD45hi), and subsets within contour plots representing (**b**) activated monocytes (Ly6ChiCCR2+), (**c**) T cells (TCRβ+), and (**d**) helper T cells (CD4+) from young and old mice. Histogram represents the respective number of cells and the calcein median fluorescence intensity for (**a**–**d**). The results were expressed as mean ± SD (*n* = 5–11 mice per group). Statistical analysis was defined by applying non-parametric Mann–Whitney test.

Fe levels influence immune functions, enhancing the proliferation and differentiation of inflammatory cells [55]. By increasing their activation, Fe regulates the production of pro-inflammatory mediators, which are subsequently released into circulation. In turn, these affect Fe metabolism by reducing Fe levels [39]. This interplay was assessed in mice, during aging, by flow cytometry. Calcein fluorescence is quenched by Fe and, therefore, used to measure intracellular Fe content in immune cells. The number of circulating activated immune cells, such as leukocytes, monocytes, and T cells, shown as examples in (Figure 4), was quantified in the peripheral blood of young vs. old mice by flow cytometry. Despite the lack of differences in total cell counts, a higher level of intracellular Fe was found in old animals. Calcein fluorescence quenching is an indirect Fe measurement, but it provides an indication of the intracellular accumulation of this metal. Nevertheless, the results were confirmed by also measuring the expression of FtH by qRT-PCR in isolated cells (Martins A.C. (2023). *Pro-inflammatory priming to the brain: the underlying cause of Parkinson’s disease.* Manuscript in preparation). Hence, these findings confirm the existence of age-related changes in peripheral immune function, which are influenced by an increased intracellular Fe content.

### 3.5. Increased Brain Permeability in Old Mice

**Figure 5:**
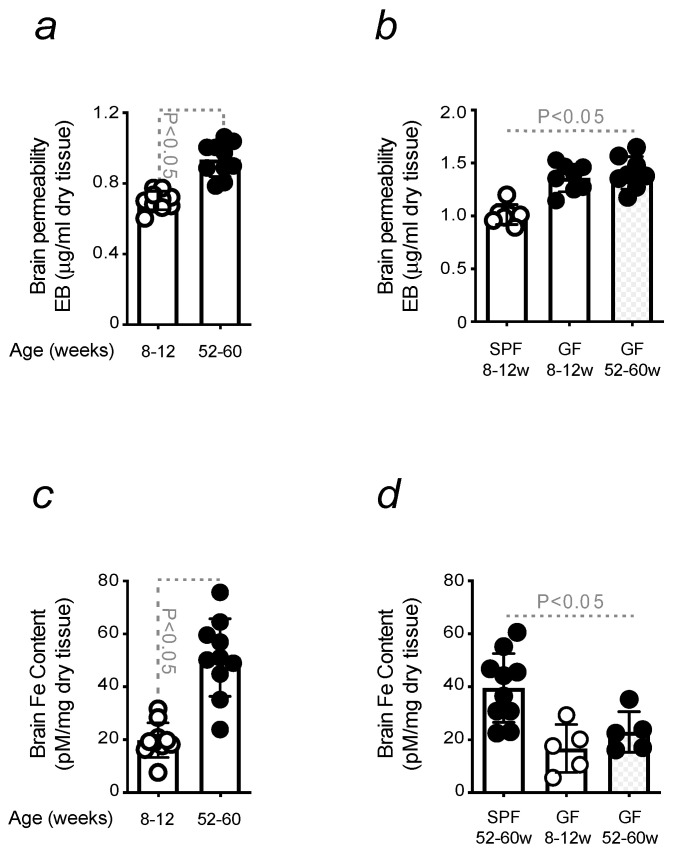
Increased brain permeability in old mice. (**a**) BBB permeability in young vs. old mice and (**b**) in GF animals colonized with stool from conventional old mice. (**c**) Brain Fe content was measured in (**a**,**d**) as in (**b**). The results were expressed as mean ± SD (*n* = 5–14 mice per group). Statistical analysis was performed by applying a parametric one-way ANOVA Test.

Data from our laboratory demonstrate that circulating Fe-loaded immune cells can cross the BBB and increase its permeability. When entering the brain, they contribute to Fe accumulation in this organ (Martins A.C. (2023). *Pro-inflammatory priming to the brain: the underlying cause of Parkinson’s disease*. Manuscript in preparation). A loss of BBB integrity was confirmed to occur during aging, as old mice presented an increased BBB permeability in relation to young animals (Figure 5a). This was also found when comparing young conventional vs. GF mice, which present an increased BBB permeability already at 8–12 weeks old. However, no significant changes were found in age-matched GF mice colonized with stool collected from conventional old animals (Figure 5b). Loss of BBB integrity favors the entry of detected Fe-loaded immune cells into the brain. Although Fe is essential for normal brain function, its excess causes oxidative damage and neuronal death [56], thus being postulated as the leading cause of neurodegeneration in both Alzheimer’s disease [57] and PD [58]. The infiltration of Fe-loaded immune cells into the brain promotes the accumulation of Fe in this organ, as observed in old mice (Figure 5c). No difference in Fe was found in young GF animals or colonized with stool from conventional old mice, as both presented a reduced level when compared to 52–60 weeks old animals (Figure 5d). Hence, the increased BBB permeability in GF mice is not accompanied by a higher level of Fe in the brain.

Overall, these results indicate that the infiltration of Fe-loaded immune cells is responsible for the accumulation of this metal in the accessed organ.

### 3.6. Reduced Neuroinflammation and Intracellular Fe Accumulation in the Brain of Old GF Mice

**Figure 6:**
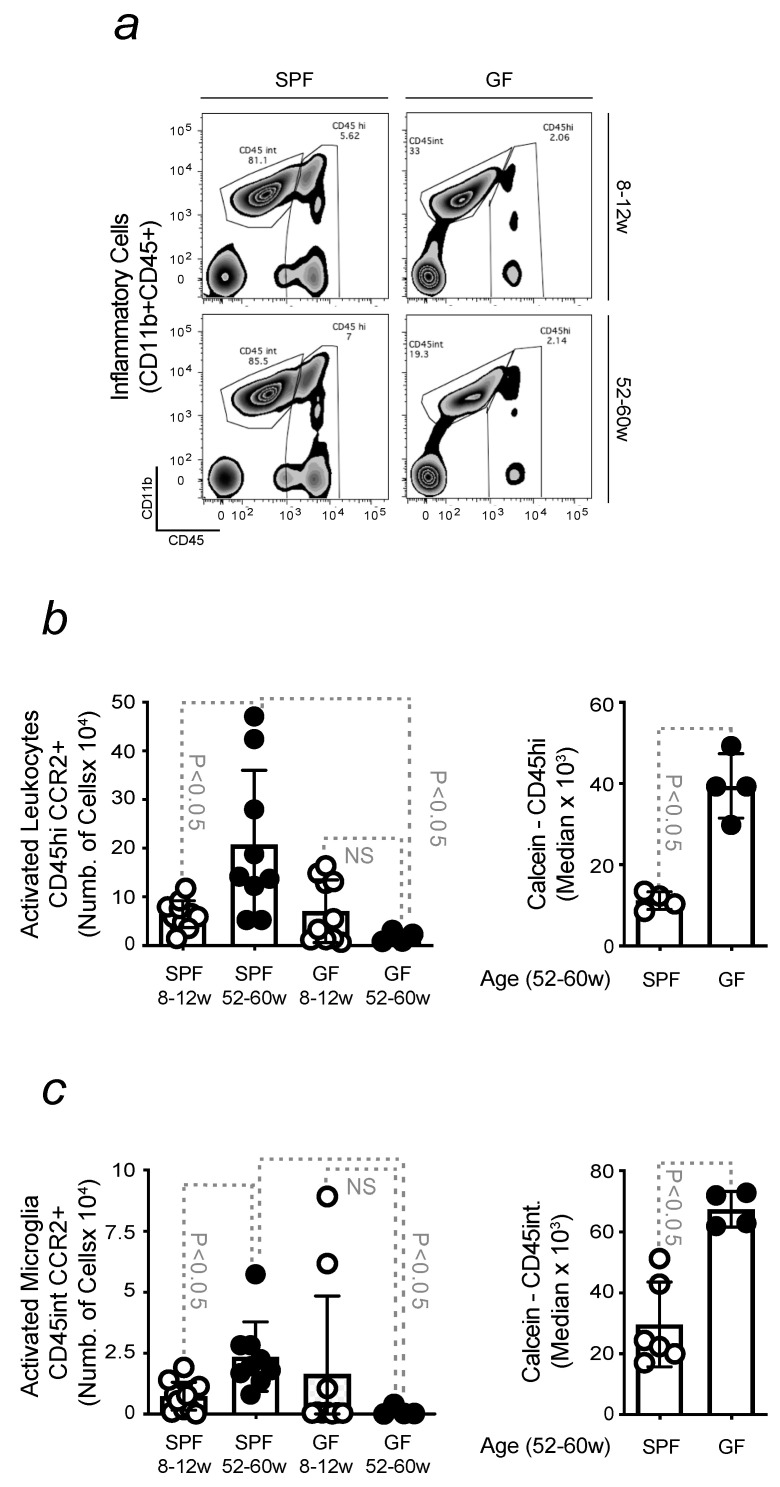
Reduced neuroinflammation and intracellular Fe accumulation in the brain of old GF mice. Gating strategy of (**a**) freshly isolated infiltrated leukocytes (CD45hi) and microglia (CD45int), within contour plots, from the brain of young vs. old conventional vs. GF mice. (**b**) Total number of activated infiltrating leukocytes in the brain of young and old mice, maintained in conventional or gnotobiotic conditions, and calcein median fluorescence intensity for CD45hi from aged animals (**c**). Total number of activated microglia in young and old mice, maintained in conventional or gnotobiotic conditions, and calcein median fluorescence intensity for CD45int from aged animals. The results were expressed as mean ± SD (*n* = 4–11 mice per group). Statistical analysis was performed by applying non-parametric Mann–Whitney Test.

To further prove the importance of the gut–brain axis in the development of a neuroinflammatory phenotype, which characterized PD, comparative analyses were conducted, by flow cytometry, in conventional and GF mice, during aging. Both young and old GF animals showed a significant decrease in the total number of infiltrated cells into the brain in relation to aged-matched conventional mice (Figure 6a). Similar data were also found when quantifying microglia counts (Figure 6a). The activation of these cells revealed a similar trend, reflecting the protective advantage of GF animals against neuroinflammation. The decreased number of infiltrated cells is also associated with a lower level of intracellular Fe (Figure 6b), as observed in microglia (Figure 6c). In agreement with these results, when compared to conventional old mice, reduced infiltration of T lymphocytes was assessed in GF during aging, as shown by the lower number and activation of helper CD4 T cells and cytotoxic CD8 T cells. No significant differences were observed during aging in GF mice (Figure S4).

Overall, these findings indicate that changes in gut microbiota, occurring physiologically during aging, trigger a sub-chronic inflammation, which compromises gut integrity and disrupts Fe homeostasis. The subsequent formation of circulating Fe-loaded cells was shown to contribute to an increase in the level of brain Fe when infiltrating into this organ. The resulting neuroinflammatory phenotype contributes to sensitizing the development of PD, thus revealing the importance of new therapies targeting the gut to protect against neuronal damage.

### 3.7. Oral Administration of the Fe Chelator Deferiprone Protects Mice against Age-Related Dysbiosis and the Development of PD

**Figure 7:**
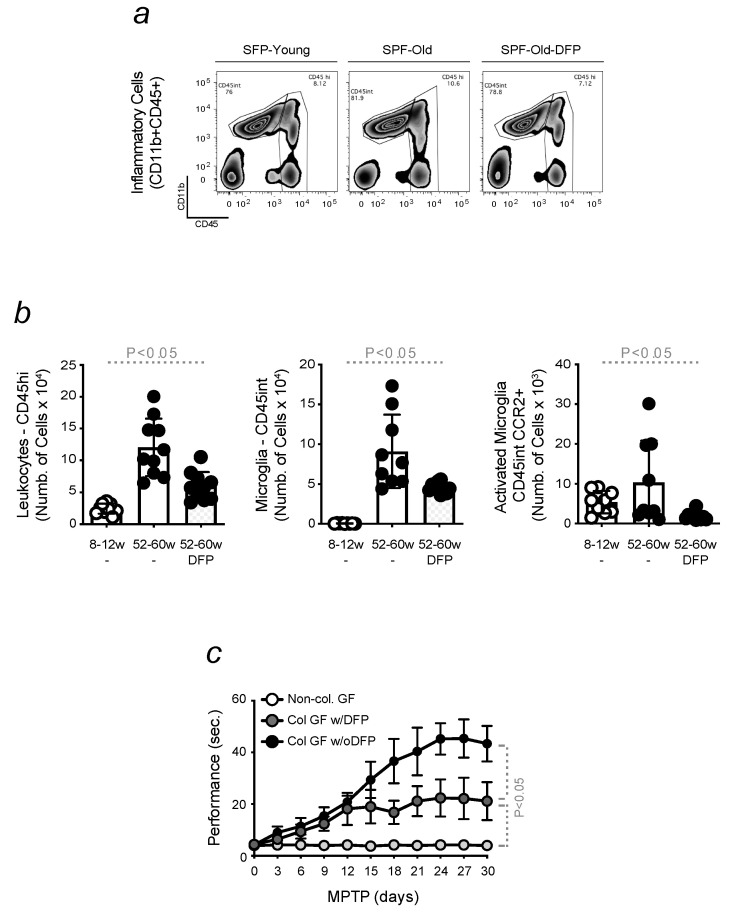
Oral administration of the Fe chelator DFP protects mice against age-related dysbiosis and the development of PD. Gating strategy of (**a**) freshly isolated infiltrated leukocytes (CD45hi) and microglia (CD45int), within contour plots, from the brain of young vs. old mice, treated or not with DFP. (**b**) Total number of cells in (**a**) and microglia activation. (**b**) The results were expressed as mean ± SD (*n* = 4–11 mice per group). Statistical analysis was performed by applying non-parametric Mann–Whitney Test. (**c**) Motor performance of GF mice, colonized or not (Ctrl) with stool samples collected from conventional old mice, treated or not with DFP (10 mg/kg, i.p. for 15 days). The results are expressed as mean ± SD (*n* = 7–10 mice per group). Statistical analysis was performed by applying the one-way ANOVA test.

The Fe chelator DFP is clinically used to ameliorate PD symptoms, given the contribution of brain Fe accumulation to neurodegeneration [59]. The positive effect of DFP on ameliorating motor function was attributed, in our experiments, to the inhibition of the neuroinflammatory phenotype associated with aging (Figure 7a,b). A decrease in the number of leukocytes and microglia, as well as their activation, was observed when old mice received a low dose of DFP for 15 days (Figure 7a,b). The same profile was observed for other inflammatory cells (Martins A.C. (2023). Pro-inflammatory priming to the brain: the underlying cause of Parkinson’s disease. Manuscript submitted). In agreement with our findings demonstrating that PD might originate in the gut due to age-related dysbiosis, we assessed whether the colonization of GF mice with stool collected from old animals treated with DFP conferred protection against PD. The results obtained showed that this was the case. A reduced locomotor dysfunction was measured in analyzed GF mice in response to MPTP. Contrarily, GF mice receiving bacterial content from conventional old animals, only treated with MPTP, showed a higher difficulty in descending the pole. Non-colonized GF animals were used as controls (Figure 7c).

Overall, these data demonstrate the efficacy of protective strategies that therapeutically target the etiology of PD rather than only treating its symptoms. This might aid in decreasing the severity of the motor dysfunction presented by PD patients.

## 4. Discussion

Aging represents a risk factor for many diseases, including neurodegenerative pathologies [60,61]. It is a physiological event characterized by the development of a systemic but sub-chronic inflammatory response, to which the composition of gut microbiota significantly contributes [62,63]. The biodiversity of gut microbes decreases during aging, altering the balance between commensal and opportunistic bacteria. This phenomenon, known as dysbiosis [64], is characterized by a reduced capacity of the gut to adapt to biological, environmental, or dietary changes, thus favoring the occurrence of low-grade intestinal inflammation [65]. The activation of immune cells depends on Fe availability, as the proliferation of potentially harmful pathogens. Hence, disruption of Fe homeostasis shifts the gut microbiome and exacerbates dysbiosis [66].

Evidence proves that age-related dysbiosis is a biomarker for PD prognostics. GI symptoms are often developed by PD patients long before the appearance of gait disturbances and postural instability. Moreover, post-mortem detection of α-syn aggregates in the gut of PD-affected individuals encouraged investigating the mechanisms through which α-syn could be transported to the brain, where it accumulates. The pathophysiology of α-syn was found to strictly depend on gut microbiota, which contributes to clearing this protein and prevents its aggregation. In transgenic mice overexpressing α-syn, intestinal pathogens were shown to play a key role in reducing the neuroinflammatory phenotype and the motor deficits that animals spontaneously developed [14,27]. Hence, the existence of bilateral communication between the central and enteric nervous systems led researchers to believe that PD originates in the intestine and then propagates to the brain through the involvement of what is referred to as the gut–brain axis [62,67].

In this study, we confirmed the importance of age-related gut disturbances for PD development [68]. Comparative analyses were conducted to evaluate motor dysfunction in conventional and GF mice. The results demonstrated that the latter are resistant to the induction of pharmacological PD. This protective effect is reverted when GF mice were colonized with stool collected from old mice maintained in the conventional environment (Figure 1).

The lack of susceptibility of GF mice was also shown by the absence of a neuroinflammatory phenotype that characterizes PD-induced mice (Supplementary Figures S1 and S2). Under the same circumstances, increased gut permeability and Fe accumulation were shown to occur (Figure 2). This protective advantage was attributed to the effects caused by age-related dysbiosis [69], namely to the inflammatory response and increased level of Fe content in the intestine. The expression of pro-inflammatory markers was higher in the gut of old mice in relation to young animals. Calprotectin, a clinical biomarker, used clinically to detect GI disturbances and found in the gut and serum of PD patients [70], was increased in old mice. This correlates with the notion that aging is, per se, an inflammatory process, also proved by the quantification of MCP-1, a chemokine responsible for recruiting immune cells to the site of inflammation [71,72]. Pro-inflammatory cytokines, such as IL-6 and TNF, are also associated with GI disturbances [73], as proved in our settings (Figure 3). Consistent with the cross-talk between inflammation and Fe metabolism [74,75] and how aging enhances tissue Fe accumulation [62,63], the content of this metal was measured in the gut of old mice. It was found higher when compared to young animals (Figure 3), an observation also confirmed by the increased expression of the Fe-storing protein FtH (Figure 3). Aging-induced regulation of intestinal Fe uptake was also demonstrated by quantifying by qRT-PCR, Fe importers DMT-1 and TfR-1, which showed levels comparable to old mice (Supplementary Figure S3).

Age-related disturbances compromise the integrity of the gut barrier and are often associated with the release of pathogens or bacterial metabolites that might reach the blood [76]. Anti-microbial effector functions are regulated by Fe, a nutrient for pathogens. Thus, it needs to be withheld to prevent their proliferation. This is a defense mechanism regulated by immune cells, which buffers Fe and restricts its availability to microbes [77]. The association of this phenomenon with aging was demonstrated in our study, showing that, despite the absence of significant differences in the number of circulating inflammatory cells, the Fe content increases with advancing age (Figure 4). Although the remodeling of Fe homeostasis proved its efficiency in protecting against organ damage, Fe-loaded immune cells become pro-inflammatory and release cytokines contributing to cross-vascular barriers [78,79,80]. The blood is a circulating compartment, and inflammatory cell extravasation can induce tissue damage, compromising organ function to the extent that relies on its sensitivity. When inflammatory cells enter the brain, they cause neuroinflammation and prime the brain to neurodegenerative disease onset (Manuscript in preparation). An increased BBB permeability was observed in old mice. This phenomenon was associated with age-related dysbiosis since no increase in BBB permeability was observed in GF mice during aging. Gut microbes are essential in BBB regulation. When compared to conventional mice, young GF animals already presented a compromised BBB (Figure 5). These results are consistent with a decreased expression of tight junction proteins in GF mice due to the function of gut microbes in preserving brain endothelium [81,82]. The impairment of BBB integrity in conventional mice was found to be associated with an increased accumulation of brain Fe, not observed in GF animals. The absence of gut microbes reduces brain Fe accumulation to values comparable to conventional young mice. The level of Fe in the brain of GF mice does not change with aging (Figure 5), as circulating inflammatory cells count infiltrating the brain of GF mice. Opposite data were found in conventional mice. According to our experimental conditions, in GF mice, these cells are Fe-spared and, therefore, less pro-inflammatory, as Fe is a known pro-oxidant [39]. This also reflects the absence of a neuroinflammatory phenotype, given that in GF mice, microglia are poorly activated when compared to conventional mice (Figure 6). Hence, it is discarded that in GF mice, Fe can fuel neuroinflammation and, consequently, neuronal death. This observation contributes to explaining the resistance of GF mice to the development of age-related neurodegenerative diseases, where inflammation and Fe are known to catalyze [83,84].

The importance of Fe in PD was also demonstrated in humans, with Fe chelation therapy being an approach used to reduce brain Fe overload. However, the effects of DFP in PD are still unclear, possibly due to the late time of administration, i.e., when patients already suffer from motor symptoms [43]. Targeting etiology rather than clinical manifestations could better protect against disease onset. Hence, we assessed whether the administration of DFP, as a protective and low-dose therapy, administered for 15 days to healthy old mice protected against the neuroinflammatory phenotype, physiologically developed with aging. A reduced number of infiltrated leukocytes was found along with lower microglia counts and activation in the brain of old mice treated with DFP when compared to young control or non-treated aged animals (Figure 7). This clearly indicates that protective strategies could be used against neurodegenerative disease onset. An early symptom of PD is GI disturbances associated with disruption of Fe metabolism. Hence, GF mice were used to further test this hypothesis, and, as such, they were colonized with stool collected from animals treated with DFP. They presented a reduced PD severity, compared to age-matched GF animals, receiving non-treated bacterial contents from old animals. These data indicate that Fe plays an important role in mediating the effects of age-related dysbiosis, proposing protective treatments as an efficient therapy to retard PD progression rather than treating its symptoms.

It is important to mention that this study presents some limitations since the experiments were conducted by following the strict procedures of gnotobiotic animal handling. Fecal contents were collected at the same hours and from aged-matched mice fed with the same diet to restrict potential parameters influencing biological variability. Although it was shown that the loss in Clostridiales and Bifidobacterium, and the enrichment in Proteobacteria and pathobionts, such as Enterobacteriaceae, characterize age-related dysbiosis [24], further studies are needed to understand the influence of other parameters. The impact of diet, circadian rhythm, and environmental conditions on the composition of intestinal microbes will provide new insights into risk factors for gut dysbiosis and disease onset. By conducting experiments in physiological circumstances and using targeted metabolomics, we might contribute to shedding light on the mechanistic aspects of the ecological risks for human health [85]. The growing use of engineered and silver nanoparticles in a variety of consumer products might represent a concern for gut dysbiosis, enhancing the incidence of chronic and progressive pathologies [24,85].

It is important to highlight, though, that clinical studies on gut microbiota in patients have been inconsistent, presumably due to a great biological variation between and within individuals. In addition, there is no consensus on the “ideal” composition of gut microbiota for optimal physical functioning and mental well-being [86]. Hence, further investigations are required to ensure that findings in animal models can be translated into humans.

## 5. Conclusions

Using PD as a model of neurodegenerative diseases, our study revealed that age-related dysbiosis and the resulting sub-chronic inflammation prime the brain to disease onset. The importance of gut microbiota composition in PD further proves the intestinal origin of this pathology. Hence, our study highlights the need to focus on therapeutic strategies against this target to reduce the priming effect on neuroinflammation and brain Fe accumulation during aging.

## Figures and Tables

**Table 1:** List of primer sequences.

Transcript	Primer Forward (5′-3′)	Primer Reverse (5′-3′)
*ArbP0*	CTTTGGGCATCACCACGAA	GCTGGCTCCCACCTTGTCT
*Gadph*	ACCACAGTCCATGCCATCAC	CACCACCCTGTTGCTGTAGCC
*Th*	GGTATACGCCACGCTGAAGG	TAGCCACAGTACCGTTCCAGA
*FtH*	CCATCAACCGCCAGATCAAC	GCCACATCATCTCGGTCAAA
*TfR-1*	TGTGACCTGTGTATTGGCCC	GCAGGGTTCTTTCCTTCGGT
*Dmt-1*	GCAGTGGTTAGCGTGGCTTATT	AGACAGACCCAATGCAATCAAA
*S100/A8*	TGTCCTCAGTTTGTGCAGAATATAAA	TCACCATCGCAAGGAACTCC
*S100/A9*	GGTGGAAGCACAGTTGGCA	GTGTCCAGGTCCTCCATGATG
*IL-6*	TAGTCCTTCCTACCCCAATTTCC	TTGGTCCTTAGCCACTCCTTC
*Tnf*	ACGGCATGGATCTCAAAGAC	AGATAGCAAATCGGCTGACG
*Mcp-1*	ACTCACCTGCTGCTACTCAT	CTACAGCTTCTTTGGGACA

## Data Availability

The data that support the findings of this study are available from the corresponding author upon reasonable request.

## Supplementary Materials

The following supporting information can be downloaded at: https://www.mdpi.com/article/10.3390/microorganisms11040880/s1, Figure S1: Age-related dysbiosis enhances PD-driven leukocytes infiltration into the brain; Figure S2: Age-related dysbiosis activates microglia in response to PD; Figure S3: Impaired intestinal Fe absorption during aging; Figure S4: Age-related dysbiosis primes the brain to neuroinflammation.
