# Supplementary material for: Gut Dysbiosis: A Target for Protective Interventions against Parkinson’s Disease
Source: Microorganisms. 2023 Mar 29;11(4):880. doi: 10.3390/microorganisms11040880 (PMC10146107; doi:10.3390/microorganisms11040880)
Supplement: Supplementary file 1 [file microorganisms-11-00880-s001.zip › microorganisms-2289104-supplementary.pdf]

Infiltrating inflammatory cells, including leukocytes, were found in the brain of PD-induced mice, and their activation was quantified by flow cytometry. An increased expression of the surface inflammatory marker, C-C chemokine receptor type 2 (CCR2), was detected in infiltrated leukocytes and quantified.

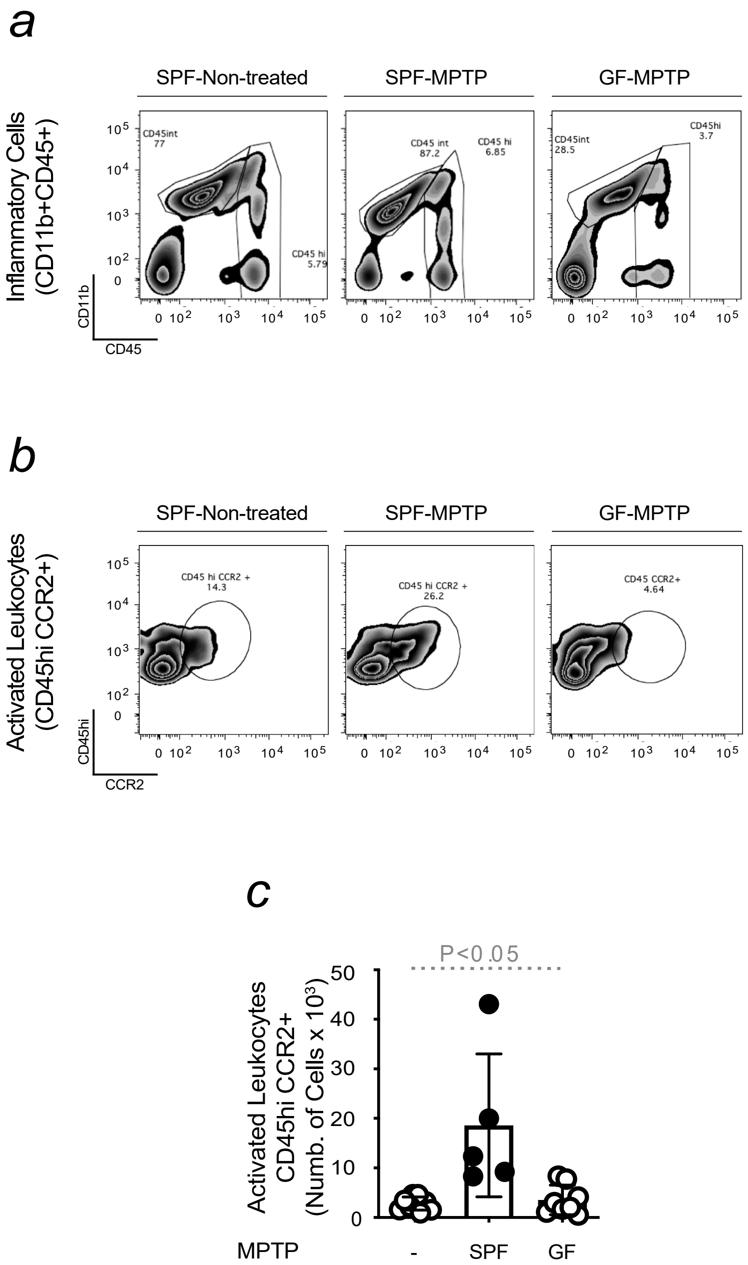

Figure S1. Age-related dysbiosis enhances PD-driven leukocyte infiltration into the brain. Gating strategy of (**a**) freshly isolated infiltrated leukocytes (CD45hi) and microglia (CD45int), within contour plots, from the brain of conventional and GF mice, exposed or not to MPTP (20 mg/kg, i.p., 4 injections 2 h apart). (**b**) Gating strategy of leukocyte activation of mice, within contour plots, as in (**a**). (**c**) Number of activated cells of mice as in *a)*. The results were expressed as mean ± SD (*n* = 5–10 mice per group). Statistical analysis was performed by applying the one-way ANOVA test.

An increased number of microglia was found in mice developing PD. Their activation, quantified by flow cytometry with the expression of the major histocompatibility complex II (MHCII), was shown to contribute to the neuroinflammatory phenotype caused by MPTP administration.

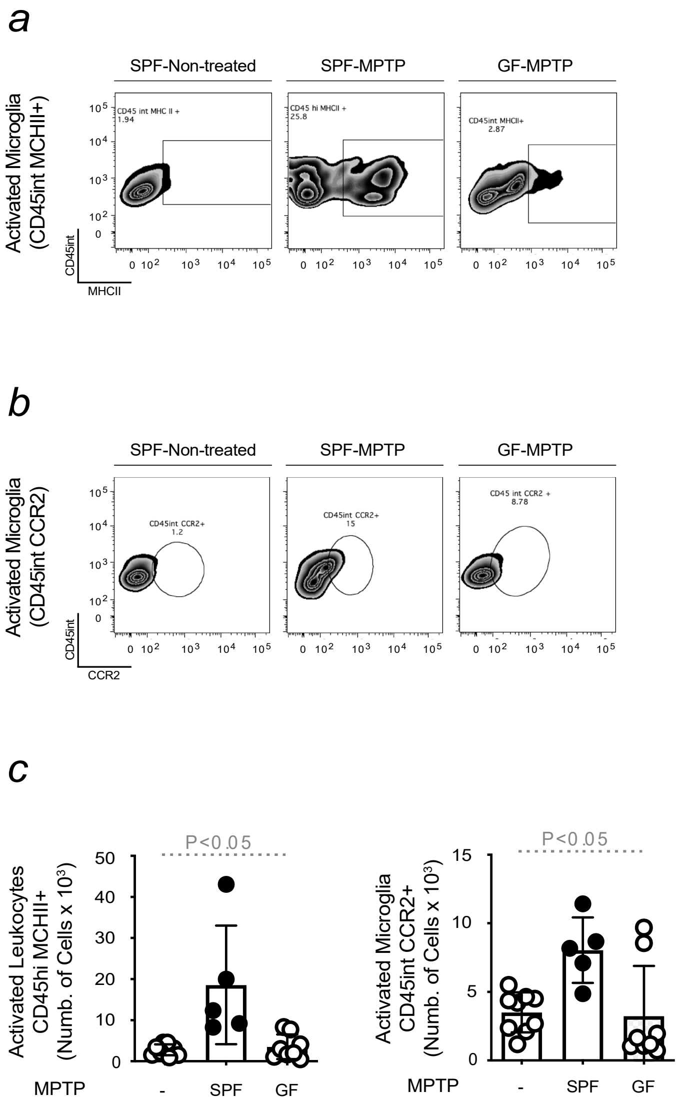

Figure S2. Age-related dysbiosis activates microglia in response to PD. Gating strategy of (**a**) freshly isolated activated microglia (CD45int MHCII+), within contour plots, from the brain of conventional and GF mice, exposed or not to MPTP (20 mg/kg, i.p., 4 injections 2 h apart). (**b**) Gating strategy of activated microglia (CD45int CCR2+), within contour plots, of mice as in (**a**). (**c**) Number of activated cells of mice as in (**a**,**b**). The results were expressed as mean ± SD (n = 5–10 mice per group). Statistical analysis was performed by applying the one-way ANOVA test.

Increased mRNA expression of DMT-1 and TFR-1 expression in the gut, these being genes involved in iron intake.

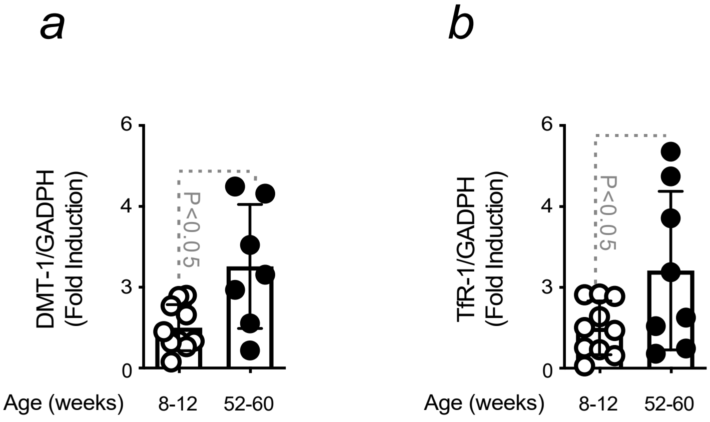

Figure S3. Impaired intestinal Fe absorption during aging. mRNA expression of (**a**) DMT-1 measured in the gut of young vs. old mice. (**b**) TfR-1 is measured as in (**a**). The results were normalized to GADPH, used as housekeeping gene, and expressed as mean ± SD (n = 10–15 mice per group). The Student's t-test was applied to define statistical differences.

Reduced infiltration of T lymphocytes assessed in young vs. old convention old and GF. A lower number and activation of helper CD4 T cells and cytotoxic CD8 T cells were observed in old GF mice when compared to conventional old animals. No significant differences were observed during aging in GF mice.

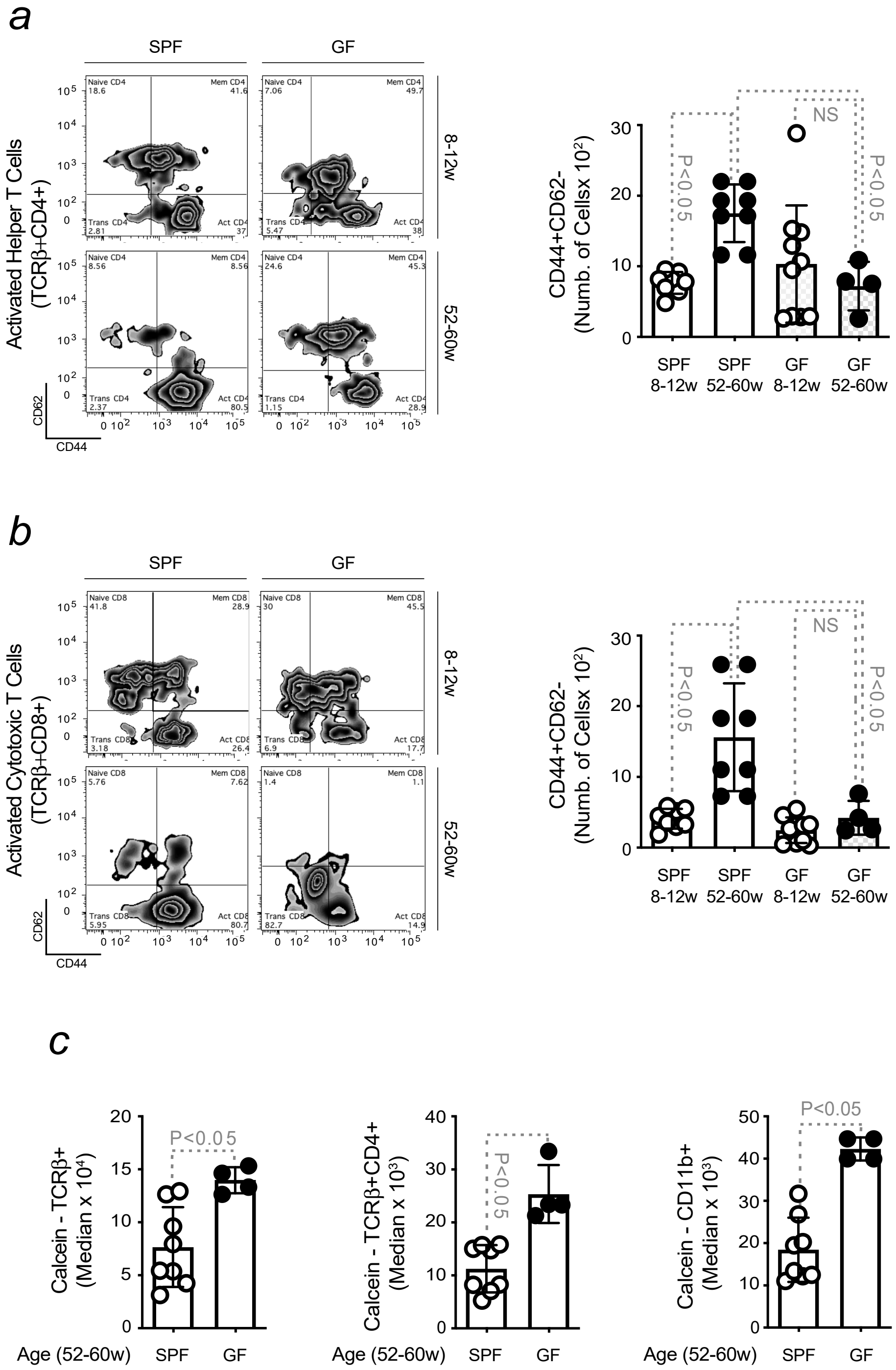

Figure S4. Impaired intestinal Fe absorption during aging. Gating strategy and quantification of (**a**) freshly isolated infiltrated Helper (CD4+) from the brain of young vs. old conventional vs. GF mice, and (**b**) cytotoxic (CD8+) T cells from mice as in (**a**), representing from the upper left quadrant, clockwise, naïve, memory, and activated cells. The results were expressed as mean ± SD (*n*=4–11 mice per group). Statistical analysis was performed by applying non-parametric Mann–Whitney Test. (**c**) Calcein median fluorescence intensity for cells from mice as in (**a**). The Student's *t*-test was applied to define statistical differences.
